# Socio-demographic features of bipolardisorder in womenin the southern region of Tunisia

**DOI:** 10.1192/j.eurpsy.2021.1649

**Published:** 2021-08-13

**Authors:** A. Kerkeni, W. Abbes, M. Nfoussi, F. Amorri, L. Ghanmi

**Affiliations:** 1 The Department Of Psychiatry, Hospital of gabes, Gabes, Tunisia; 2 Outpatients Department, Hospital of gabes, Gabes, Tunisia

**Keywords:** bipolar disorder, women, Socio-demographic features

## Abstract

**Introduction:**

Bipolar disorder (BD) is a common and disabling condition. Gender differences are potentially important and can manifest in many ways.

**Objectives:**

To determine the socio-demographic characteristics of women with BD, followed at the department of psychiatry of Gabes (southern of Tunisia).

**Methods:**

A retrospective descriptive and analytical study was undertaken including all the patients having consulted for the first time in the department of psychiatry of Gabes, from January 1st, 2010 to December 31, 2016, for whom the diagnosis of a bipolar disorder was established according to the DSM-IV criteria. Sociodemographic and clinical data were assessed. Patients were divided into two groups according to gender. The collected data was compared between the two groups. The statisticalanalysiswasexecuted on the software SPSS (20thedition).

**Results:**

We included 193 patients with BD (women = 103). The mean age of the women studied was 39.9 years. Women with BD had the following characteristics: married (55.3%), unemployed (65.1%), having an urban origin (75.7%), attending the primary or secondary school level (76.7%) and with an middle socioeconomic level (62.1%). Among the women studied, 9 (8.7%) were smokers, 2 (1.9%) consumed alcohol, and one (0.9%) used cannabis. Regarding the socio-demographic differences by gender, bipolar women were significantly less professionally active (p<10-3), less educated (p= 0.009), more frequently married, widowed or divorced (p <10-3) and having dependent children (p=0.008).

**Conclusions:**

Our study made it possible to note the socio-demographic particularities of the woman followed for BD. A better knowledge of these particularities is the best guarantee of adequate care.

**Disclosure:**

No significant relationships.

